# Benign Tumors in Long-Term Survivors of Retinoblastoma

**DOI:** 10.3390/cancers13081773

**Published:** 2021-04-08

**Authors:** Milo van Hoefen Wijsard, Sara J. Schonfeld, Flora E. van Leeuwen, Annette C. Moll, Armida W. Fabius, David H. Abramson, Johanna M. Seddon, Jasmine H. Francis, Margaret A. Tucker, Ruth A. Kleinerman, Lindsay M. Morton

**Affiliations:** 1 Department of Ophthalmology, Amsterdam UMC, Vrije Universiteit Amsterdam, Cancer Center Amsterdam, 1081 HV Amsterdam, The Netherlands; m.vanhoefenwijsard@amsterdamumc.nl (M.v.H.W.); a.moll@amsterdamumc.nl (A.C.M.); a.fabius@amsterdamumc.nl (A.W.F.); 2 Division of Cancer Epidemiology and Genetics, National Cancer Institute, Bethesda, MD 20892, USA; schonfes@mail.nih.gov (S.J.S.); tuckerp@mail.nih.gov (M.A.T.); kleinerr@mail.nih.gov (R.A.K.); 3 Department of Epidemiology, The Netherlands Cancer Institute, 1066 CX Amsterdam, The Netherlands; f.v.leeuwen@nki.nl; 4 Memorial Sloan Kettering Cancer Center, New York, NY 10065, USA; Abramsod@mskcc.org (D.H.A.); francij1@mskcc.org (J.H.F.); 5 Department of Ophthalmology and Visual Sciences, University of Massachusetts Medical School, Worcester, MA 01605, USA; jseddon@earthlink.net

**Keywords:** retinoblastoma, subsequent benign tumor, lipoma, *RB1*, leiomyoma, cumulative incidence, retinoblastoma survivor, epidemiology, hereditary retinoblastoma, subsequent malignant neoplasms

## Abstract

**Simple Summary:**

It is well-established that hereditary retinoblastoma survivors have a substantially increased risk of developing subsequent malignant neoplasms (SMNs). Although clinicians have long suspected that this population is also at increased risk for developing benign neoplasms, the evidence is unclear. Benign tumors can substantially impact health status and quality of life, while raising questions for clinicians, when faced with a mass in a retinoblastoma survivor. By 60 years following retinoblastoma diagnosis, 17.6% of hereditary survivors had developed a benign tumor, with lipomas and leiomyomas being the most frequently diagnosed types. Additionally, we report both an increased risk of benign tumors after SMNs and a reciprocal increased risk of SMNs after benign tumors among hereditary retinoblastoma survivors. If confirmed, the large magnitude of the absolute risks and the association between benign tumors and SMNs in this population may have implications for long-term surveillance.

**Abstract:**

Hereditary retinoblastoma survivors have substantially increased risk of subsequent malignant neoplasms (SMNs). The risk of benign neoplasms, a substantial cause of morbidity, is unclear. We calculated the cumulative incidence of developing benign tumors at 60 years following retinoblastoma diagnosis among 1128 hereditary (i.e., bilateral retinoblastoma or unilateral with family history, mutation testing was not available) and 924 nonhereditary retinoblastoma survivors diagnosed during 1914–2006 at two US medical centers with follow-up through 2016. Using Cox proportional hazards regression, we compared benign tumor risk by hereditary status and evaluated the association between benign tumors and SMNs. There were 100 benign tumors among 73 hereditary survivors (cumulative incidence = 17.6%; 95% confidence interval [CI] = 12.9–22.8%) and 22 benign tumors among 16 nonhereditary survivors (cumulative incidence = 3.9%; 95%CI = 2.2–6.4%), corresponding to 4.9-fold (95%CI = 2.8–8.4) increased risk for hereditary survivors. The cumulative incidence after hereditary retinoblastoma was highest for lipoma among males (14.0%; 95%CI = 7.7–22.1%) and leiomyoma among females (8.9%; 95%CI = 5.2–13.8%). Among hereditary survivors, having a prior SMN was associated with 3.5-fold (95%CI = 2.0–6.1) increased risk of developing a benign tumor; the reciprocal risk for developing an SMN after a benign tumor was 1.8 (95%CI = 1.1–2.9). These large-scale, long-term data demonstrate an increased risk for benign tumors after hereditary versus nonhereditary retinoblastoma. If confirmed, the association between benign tumors and SMNs among hereditary patients may have implications for long-term surveillance.

## 1. Introduction

Retinoblastoma is the most common primary intraocular malignancy in infancy and childhood. Patients with hereditary retinoblastoma carry a germline mutation in the *RB1* gene and are at substantially increased risk of subsequent malignant neoplasms (SMNs) compared to patients with nonhereditary retinoblastoma and to the general population [1,2,3,4]. Due to excellent survival following retinoblastoma, SMNs are now the leading cause of death in patients with hereditary retinoblastoma in economically advanced countries [5,6].

Less is known about the occurrence of subsequent benign neoplasms in retinoblastoma survivors. Previous reports, including one from our cohort, showed an excess of lipoma in patients with hereditary retinoblastoma compared to nonhereditary survivors [7,8,9], suggesting that germline *RB1* mutations may also contribute to benign tumor development. However, little is known about the occurrence of other types of benign tumors, and risk factors have not been evaluated, in part due to the fact that benign tumors are not reportable to most cancer registries and thus are not systematically ascertained by most cohorts. Nevertheless, lipomas can affect quality of life [10,11], uterine leiomyomas are responsible for heavy and prolonged menstrual bleeding and fertility issues [12], and in survivors of childhood cancer other than retinoblastoma, subsequent benign meningiomas have been shown to cause significant neurologic morbidity and even mortality [13]. Thus, benign tumors can substantially impact health status and quality of life, while raising questions for clinicians when faced with a mass in a retinoblastoma survivor.

To improve our understanding of benign tumor occurrence among retinoblastoma survivors, we evaluated the risk for benign tumors in the National Cancer Institute Long-Term Follow-up Study of Retinoblastoma Survivors and whether the occurrence of benign tumors is associated with the occurrence of SMNs.

## 2. Methods

### 2.1. Study Population and Data Collection

A total of 2136 retinoblastoma patients, treated during 1914–2006 at two major medical centers in New York, NY and Boston, MA, were identified from medical records, as previously described in detail [1,5]. Exclusion of non-US residents (*n* = 45), survivors who were first seen at a study center more than five years after initial retinoblastoma diagnosis (*n* = 29), and patients with insufficient information (e.g., missing date of birth or no follow-up, *n* = 10), resulted in an eligible study population of 2052 survivors.

Abstraction of detailed data for retinoblastoma diagnosis, treatment, and family history of retinoblastoma at time of diagnosis was performed in 1984, 1996 and 2006 for patients diagnosed from 1914–1984, 1985–1996, and 1997–2006 respectively. Based on medical record data at the time of retinoblastoma diagnosis, 1128 patients with bilateral disease or unilateral disease and a positive family history were classified as having hereditary retinoblastoma whereas the remaining 924 unilateral patients with no known family history at the time of retinoblastoma diagnosis were classified as nonhereditary. Mutation testing was not available in these patients.

The study was performed in accordance with the Declaration of Helsinki and was approved by the Special Studies Institutional Review Board at the National Cancer Institute, including informed consent for questionnaires from survivors or their legal guardians for survivors < 18 years of age.

### 2.2. Subsequent Neoplasm Ascertainment

Subsequent (i.e., after treatment for retinoblastoma) benign and malignant neoplasms were systematically ascertained through initial medical record review, periodic questionnaires administered to the cohort, and linkage with the National Death Index (NDI) up until 2016. Overall, approximately 63% of the cohort responded to at least one questionnaire, with a slightly higher proportion among nonhereditary (68.2%) than hereditary (59.0%) survivors. We restricted the present analyses to incident (not ascertained by NDI) neoplasms since benign tumors are unlikely to be ascertained through death certificates. Self-reported subsequent neoplasms were included in the analyses if they were confirmed by pathology reports.

Subsequent neoplasms were classified according to the International Classification of Diseases for Oncology (third edition) [14]. Neoplasms of uncertain behavior and in situ tumors were excluded from analysis. Benign skin nevi also were excluded because of inconsistent patient reporting. Benign tumors were classified by location, considering all neoplasms in the head and neck area to be within the field of external beam radiation therapy. SMNs were further categorized by type into soft tissue (the most common subsequent neoplasm type) or non-soft-tissue neoplasms based on ICD-O-3 morphology and topography codes [15].

### 2.3. Statistical Analysis

For all analyses, individuals were followed from-the date of retinoblastoma diagnosis until the earliest of an event (first benign tumor diagnosis or first SMN for analyses evaluating risk of SMN) or censored at the date of last contact which was defined as the date of last questionnaire or, for those individuals who never completed a follow-up questionnaire, the date of medical record abstraction or death (whichever occurred first).

Cumulative incidence, accounting for the competing risk of death, was calculated for benign tumors overall and for specific tumor types with at least five cases in either hereditary or nonhereditary survivors (Stata, version 14; StataCorp, College station, TX, USA) [16]. Subtype-specific analyses further accounted for competing risks from other subtypes. Among hereditary retinoblastoma survivors, we used Cox proportional hazards regression with time since retinoblastoma diagnosis as the time scale to examine treatment and demographic factors associated with benign tumors (hazard ratios [HRs] and corresponding 95% confidence intervals [CIs]). Year and age at retinoblastoma diagnosis, sex, family history of retinoblastoma, and treatment for retinoblastoma were included as covariates in multivariable models. There were too few benign tumors reported among nonhereditary survivors to examine these factors.

Since retinoblastoma is diagnosed mainly in infants, attained age is closely related to time since retinoblastoma diagnosis and therefore not adjusted for in the Cox model. Separate Cox models were constructed for benign subtypes with >10 cases. To examine the association between malignant and benign tumors, occurrence of a prior or concurrently diagnosed malignancy was included as a time-dependent variable in the analysis of benign tumors while occurrence of a prior or concurrently diagnosed benign tumor was included as a time-dependent variable in the analysis of SMNs. Analyses were conducted using SAS software (version 9.4; SAS Institute, Cary, NC, USA) and SPSS Statistics (version 24; IBM, Armonk, NY, USA).

## 3. Results

Demographic and clinical characteristics of the cohort are listed in Table 1. Compared to nonhereditary survivors, retinoblastoma was diagnosed at a younger age in hereditary survivors and treatment mostly consisted of radiotherapy (85.9%, with or without chemotherapy), whereas the majority of nonhereditary retinoblastoma survivors (68.8%) underwent surgical treatment alone.

### 3.1. Incidence of Benign Tumors

**Figure 1:**
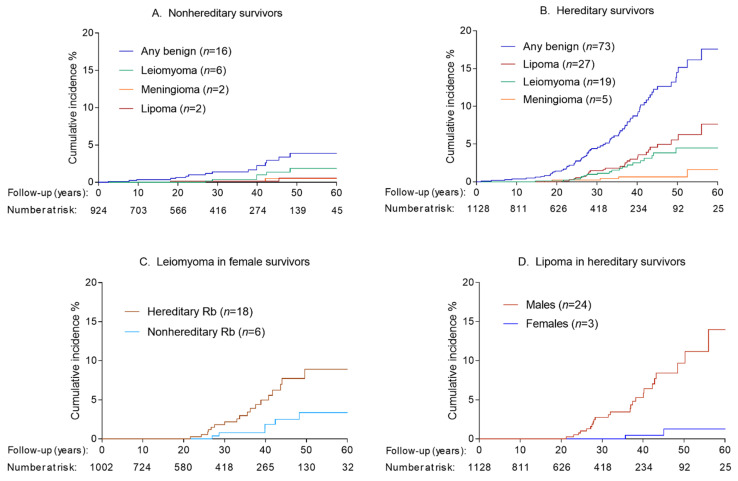
Cumulative incidence of first benign tumors in retinoblastoma survivors by time since retinoblastoma diagnosis. Patients were followed until first benign tumor diagnosis or date of last questionnaire (patients who never completed a questionnaire were censored at the earliest of death or date of medical record abstraction). All analyses account for competing risk of death. Type-specific analyses further accounted for competing risk from diagnosis of another type.

During a median follow-up of 29.4 years (range: 0 to 88.8 years), we identified 22 benign tumors among 16 nonhereditary retinoblastoma survivors. The most common types were leiomyoma (*n* = 8, 36.4%), lipoma (*n* = 4, 18.2%), and osteochondroma (*n* = 4, 18.2%) (Supplemental Table S1). Cumulative incidence for developing a benign tumor in nonhereditary retinoblastoma survivors at 60 years follow-up was 3.9% (95%CI = 2.2% to 6.4%) (Figure 1A). The 60-year cumulative incidence was highest for leiomyoma (1.9%; 95%CI = 0.7% to 4.0%).

During a median follow-up of 24.8 years (range: 0 to 75.1 years), 100 benign tumors were reported among 73 hereditary retinoblastoma survivors. Lipoma (*n* = 46, 46.0%), leiomyoma (*n* = 22, 22.0%), meningioma (*n* = 6, 6.0%), fibroma (*n* = 5, 5.0%), and benign breast tumors (*n* = 5, 5.0%) were the most frequently reported benign tumors (Table 2). Nineteen patients were diagnosed with two or more benign tumors during follow-up, with multiple lipomas occurring most often. The majority (76.7%) of the first benign tumors observed were within soft tissue. Cumulative incidence for developing a benign tumor at 60 years after retinoblastoma was 17.6% (95%CI = 12.9% to 22.8%), with the highest cumulative incidence observed for lipomas (7.6%; 95%CI = 4.4% to 12.0%) followed by leiomyomas (4.5%; 95%CI = 2.6% to 7.1%) and meningiomas (1.6%; 95%CI = 0.4% to 4.7%; Figure 1B). As illustrated in Table 2, we missed few subtype-specific cases by following patients only to their first benign tumor for analyses estimating cumulative incidence and HRs as the morphology of the first and subsequent benign tumor were the same in the vast majority of cases.

Among female hereditary survivors, the cumulative incidence for developing a leiomyoma was 8.9% (95%CI = 5.2% to 13.8%; Figure 1C) at 60 years after retinoblastoma. All cases of leiomyoma in this group were uterine whereas the one case among males occurred in the orbit. For lipoma, the 60-year cumulative incidence was much higher among male hereditary survivors (14.0%; 95%CI = 7.7% to 22.1% (*n* = 24)) compared to female survivors (1.2%; 95%CI = 0.2% to 4.2% (*n* = 3); Figure 1D). At 60 years following retinoblastoma, the cumulative incidence of head/neck (*n* = 16; in-field) lipoma was 5.1% (95%CI = 2.4% to 9.5%) and that for body and extremities (*n* = 12; out-of-field) was 2.5% (95%CI = 1.3% to 4.4%).

### 3.2. Risk Factors for Benign Tumors

In univariate analysis, hereditary retinoblastoma survivors had nearly fivefold increased risk of benign tumors compared with nonhereditary survivors (HR = 4.9; 95%CI = 2.8 to 8.4). Among hereditary retinoblastoma survivors, we examined risk factors for any benign tumor and the two most common benign subtypes (based on follow-up to the first benign tumor of any kind). In multivariable analyses, none of the demographic factors were statistically significantly associated with a higher risk of developing a benign tumor overall (Table 3). However, consistent with the cumulative incidence estimates, compared with males, female patients had one-eighth the risk of lipoma (HR = 0.1, 95%CI = 0.0 to 0.4) but 21-times the risk of leiomyoma (HR = 21.1, 95%CI = 2.8 to 161.0). Although treatment was not statistically significantly associated with benign tumors (any, lipoma or leiomyoma) among hereditary retinoblastoma survivors, we observed a 2.7-fold increased risk (95%CI = 0.9 to 8.0) of leiomyoma after combined treatment with chemotherapy with radiotherapy compared with radiotherapy alone. Notably, radiotherapy (compared with no radiotherapy) was not significantly associated with risk of head/neck lipoma (HR = 1.2; 95%CI = 0.3 to 5.7; data not shown).

### 3.3. SMNs and Subsequent Risk for Benign Tumors

We identified 252 hereditary retinoblastoma survivors who developed ≥ one incident SMN during follow-up. Nineteen hereditary survivors (26%) with a benign tumor had a prior (*n* = 16) or concurrent (*n* = 3) SMN. In multivariable analyses, a prior/concurrent SMN diagnosis was associated with a higher risk of any benign tumor (HR = 3.5; 95%CI = 2.0 to 6.1; Table 3). In additional analyses by time since SMN diagnosis, risk for developing a benign tumor was highest within the first year after SMN diagnosis (HR = 12.2; 95%CI = 5.1 to 29.0), nonsignificantly elevated 1–5 years (HR = 2.1; 95%CI = 0.7 to 6.9) and again significantly elevated at ≥5 years (HR = 2.8; 95%CI = 1.4 to 5.7). A prior or concurrent SMN was associated with a higher risk of both lipoma (HR = 3.6; 95%CI = 1.5 to 8.7) and leiomyoma (HR = 6.4; 95%CI = 2.2 to 18.6). Risk for any benign tumor did not appear to vary whether prior SMN was of a soft tissue type (HR = 3.6; 95%CI = 1.6 to 8.1) or non-soft tissue type (HR = 3.5; 95%CI = 1.8 to 6.6). We also noted similarly elevated risks of lipomas and leiomyomas by prior/concurrent SMN type (data not shown).

### 3.4. Benign Tumors and Subsequent Risk for SMNs

Twenty of the 252 hereditary retinoblastoma survivors who developed an incident SMN (7.9%) during follow-up had a benign tumor at or before SMN diagnosis. Multivariable Cox regression revealed that a prior benign tumor was associated with a higher risk of developing an SMN (HR = 1.8; 95%CI = 1.1 to 2.9). Secondary analysis showed this risk was only significant within the first year after benign tumor diagnosis (HR = 6.0; 95%CI = 2.2 to 16.3) and not thereafter [1–5 years, HR = 1.6 (95%CI = 0.6 to 4.3); ≥5 years, HR = 1.5 (95%CI = 0.8 to 2.7)].

## 4. Discussion

Using large-scale, long-term follow-up data from a well-characterized clinical cohort, we present novel data showing that hereditary retinoblastoma survivors have a 4.9-fold increased risk of benign tumors compared to nonhereditary retinoblastoma survivors. Our results are consistent with the long-presumed heightened risk of benign subsequent tumors in this population, which was previously reported only in small case series or individual reports, and likely indicate the involvement of germline *RB1* mutations in benign tumor development in these patients. We report an estimated cumulative incidence of 17.6 for developing a benign tumor at 60 years following hereditary retinoblastoma diagnosis, with lipoma and leiomyoma accounting for the largest risks. Female survivors had a markedly lower risk of lipoma compared with male survivors, but a statistically significantly higher risk of leiomyoma (mainly occurring in the uterus). Moreover, we found that occurrence of benign tumors was associated with the occurrence of SMNs in hereditary patients.

Although benign tumor occurrence may have important clinical impact for hereditary retinoblastoma survivors, our estimated cumulative incidence is somewhat lower than that recently reported among survivors of childhood cancers other than retinoblastoma in the Netherlands which estimated a 30-year cumulative incidence of 7.3% (95%CI = 5.8% to 9.1%) among individuals diagnosed with a first primary childhood cancer during 1963–1984 [17]. The corresponding estimate in our cohort at 30 years is 4.4% (95%CI = 3.1 to 6.1). It is unclear whether the lower cumulative incidence reported here reflects differences in susceptibility to and/or surveillance for benign tumors among retinoblastoma versus other childhood cancer survivors or methodologic differences in the studies. Notably, in the Dutch cohort, meningioma represented the most common benign tumor after childhood cancer, mainly after high-dose, full-volume cranial radiotherapy [17,18]; however, similar treatments are uncommon for retinoblastoma. Additionally, whereas that study based ascertainment on linkage with a database containing results from all pathology examinations in the Netherlands, our study largely relied on directly surveying survivors, with subsequent confirmation of self-reported benign tumors through pathology report review (i.e., from biopsies).

Consistent with SMN findings in hereditary retinoblastoma survivors [19], tumors of soft tissue origin were the most common type of benign tumor. We report a cumulative incidence of 7.6% for lipoma in hereditary survivors at 60 years, confirming our previous findings of heightened prevalence of lipoma in this group [7] and substantiating reported lipoma occurrence in several families and incident cases with hereditary retinoblastoma [8,9]. Previous cytogenetic analyses have shown *RB1* loss in lipoma [20,21,22], providing support for an association between germline *RB1* mutations in hereditary retinoblastoma and a high incidence of lipoma in these survivors. In our study, more than a third of hereditary survivors with a lipoma developed multiple lipomas, consistent with results from a literature review of multiple lipomas after retinoblastoma [21]. Although incidence and prevalence of lipoma is poorly reported in the literature [23], our observation of higher lipoma risk among males versus females is consistent with previous reports suggesting that lipoma occurs more frequently in men, also noted in a general population case series in which 57/66 (86%) cases of dysplastic lipoma were found in men [24]. In our study, lipoma was the only frequent benign tumor that occurred both in and outside of the radiotherapy field, but we did not observe a significant association between radiotherapy and head-and-neck lipoma.

Leiomyoma was the second most commonly reported benign tumor in hereditary retinoblastoma survivors in our study. All but one were uterine leiomyomas, which explains the high hazard ratio for leiomyoma for women compared to men and a cumulative incidence of 8.9% in female hereditary survivors at 60 years. It is uncertain how this compares to the general population as previous studies have mainly been conducted in symptomatic women or women undergoing hysterectomy. A cross-sectional study of 1756 women with uterine-related symptoms reported leiomyomas ranging from 12–24% [25]. A striking risk for malignant uterine leiomyosarcoma has previously been reported among hereditary survivors in our cohort [26]. During follow-up for this study, only one female hereditary survivor was diagnosed with a leiomyoma and a leiomyosarcoma of pelvic origin. In general, it appears that benign uterine leiomyomas do not progress into leiomyosarcoma [27]. Although our data do not provide any evidence for an association between leiomyoma and leiomyosarcoma, this is difficult to study because previously most retinoblastoma survivors in our cohort diagnosed with symptomatic uterine leiomyomata underwent hysterectomy [26]. Finally, we note that chemotherapy was associated with a nonsignificantly increased risk for the development of leiomyoma. Previously, we reported significantly higher risks of leiomyosarcoma among patients who received alkylating agent chemotherapy in addition to radiotherapy versus those who received radiotherapy alone [1], thus additional investigation of the role of chemotherapy in leiomyoma is warranted.

Benign meningioma was diagnosed in five hereditary survivors, resulting in a cumulative incidence of 1.6% at 60 years. Meningiomas may have been underascertained in this study, given that a substantial proportion of benign meningioma cases are based only on diagnostic imaging and lack histological confirmation (50% in a recent study based on cancer registry data in the US) [28]. Although radiotherapy for childhood cancer is a well-established risk factor for meningioma [18,29,30] and all five hereditary survivors with a meningioma in this study received external beam radiotherapy for their retinoblastoma, the small sample size precluded detailed assessment of meningioma risk factors in this study.

A prior or concurrent SMN was associated with a higher risk for benign tumors. The risk was the highest in the first year after SMN diagnosis, suggesting that heightened surveillance may in part explain this increase. However, the threefold elevated risk observed after five years suggests that surveillance is not the only factor. We found that a prior benign tumor was also associated with higher risk for SMNs in hereditary survivors, but this was only statistically significant during the first year following benign tumor diagnosis.

The challenge of studying subsequent benign tumors in a sufficiently large population of retinoblastoma survivors lies in the systematic ascertainment. Benign tumors are not reportable to most cancer registries nor are they likely to be a major cause of death. By directly surveying survivors, followed by pathology report confirmation, we systematically ascertained benign tumors in this population. We sought to reduce the potential for spurious findings due to heightened medical surveillance by limiting our analyses to systematically ascertained confirmed cases. Nonetheless, some limitations of our study warrant consideration. In general, over 10% of patients with sporadic unilateral retinoblastoma have a germline *RB1* mutation [31]. Because mutation testing was not available, some unilateral hereditary patients may be incorrectly considered as nonhereditary in this study, which may have inflated our risk estimates for nonhereditary retinoblastoma survivors. However, it is also possible that health care providers and hereditary survivors, knowing their elevated cancer risk, may be more inclined to investigate a less suspicious (benign) tumor leading to more histologically confirmed tumors in this group compared with nonhereditary survivors. Irrespective of hereditary status, ascertainment is probably incomplete because we largely relied on patient-report and pathology report confirmation, thus un-biopsied or asymptomatic benign tumors would have been missed.

## 5. Conclusions

In summary, hereditary retinoblastoma survivors have a significantly higher incidence of benign tumors than nonhereditary survivors, with lipomas and leiomyomas being the most frequently diagnosed benign tumor types. We noted a striking predominance of lipoma in men and leiomyoma in women. In contrast to SMNs [1,2,15], these data do not point to a major role for retinoblastoma treatment in the risk of subsequent benign tumors. Our findings suggest that there is an association between the occurrence of benign and malignant subsequent tumors among hereditary patients, but more data are needed to understand this association and the potential implications for long-term surveillance.

## Figures and Tables

**Table 1:** Characteristics of 2052 retinoblastoma survivors.

	Hereditary * Retinoblastoma		Nonhereditary Retinoblastoma	
	*N*	%	*N*	%
**Total**	1128	100%	924	100%
**Laterality**				
Unilateral	29	2.6%	924	100%
Bilateral	1099	97.4%	0	0%
**Sex**				
Male	578	51.2%	472	51.1%
Female	550	48.8%	452	48.9%
**Age at retinoblastoma diagnosis**				
0–11 months	661	58.6%	200	21.6%
12–23 months	303	26.9%	268	29.0%
≥24 months	164	14.5%	456	49.4%
**Calendar year of retinoblastoma diagnosis**				
1914–1959	304	27.0%	212	22.9%
1960–1969	297	26.3%	224	24.2%
1970–1979	248	22.0%	204	22.1%
1980–2006	279	24.7%	284	30.7%
**Family history of retinoblastoma**				
Yes	237	21.0%	0	0.0%
No/unknown	891	79.0%	924	100.0%
**Treatment for retinoblastoma**				
Surgery only	90	8.0%	636	68.8%
Radiotherapy, no chemotherapy	534	47.3%	97	10.5%
Radiotherapy and chemotherapy	435	38.6%	86	9.3%
Chemotherapy, no radiotherapy	38	3.4%	58	6.3%
Other/unknown	31	2.7%	47	5.1%

* Hereditary status was defined based on data collected from medical records at the time of retinoblastoma diagnosis. All patients with bilateral disease or unilateral disease and a positive family history (excluding offspring of the patients) were classified as having hereditary retinoblastoma; all other unilateral patients were classified as nonhereditary.

**Table 2:** Patterns of benign tumors * among hereditary retinoblastoma survivors who reported at least one benign tumor during follow-up.

First Benign Tumor	Second Benign Tumor	Subsequent Benign Tumors	*N* People
Lipoma			16
Lipoma	Lipoma		6
Lipoma	Lipoma	3rd Lipoma	2
Lipoma	Lipoma	3rd & 4th Lipoma	1
Lipoma	Lipoma	3rd, 4th & 5th Lipoma	1
Lipoma	Neurilemoma		1
Leiomyoma			17
Leiomyoma	Leiomyoma		2
Meningioma			4
Meningioma	Meningioma		1
Fibroma			2
Fibroma	Fibroma		1
Fibroma	Breast (noninvasive)		1
Breast (noninvasive)			4
Thyroid adenoma	Leiomyoma		1
Thyroid adenoma	Lipoma		1
Sebaceous adenoma			1
Cutaneous adnexal tumor	Lipoma		1
Hemangioma	Hemangioma		1
Astrocytoma			1
Sinus myxoma			1
Lymphangioma			1
Hemangioma			1
Fibrous histiocytoma			1
Ovarian adenoma			1
Tongue papilloma			1
Colorectal, unspecified			1
Head and neck, unspecified			1

* Morphology codes for 100 benign tumors in hereditary retinoblastoma survivors included 46 lipomas (ICD-O-3 morphology: 8850, 8857, 8861), 22 leiomyomas (8890), six meningiomas (9530–9531), five fibromas (8810, 8832, 9010, 9540), five breast (M-8503, 9010, 9020), two thyroid adenomas (8330), three hemangiomas (9120), one sebaceous adenoma (8410), one astrocytoma (9400), one sinus myxoma (8840), one lymphangioma (code not specified), one fibrous histiocytoma (8830), one ovarian adenoma (8440), one tongue papilloma (8050), one neurilemoma (9560), one cutaneous adnexal tumor (8407), one head and neck, unspecified (8000), and one colorectal (unspecified).

**Table 3:** Risk factors for benign tumors among 1128 hereditary retinoblastoma survivors.

		Any Benign		Lipoma		Leiomyoma	
	*N* Total	*N* Cases	HR (95% CI) ^‡^	*N* Cases	HR (95% CI) ^‡^	*N* Cases	HR (95% CI) ^‡^
**Total**	1128	73		27		19	
**Sex**							
Male	578	35	1.0 (Ref)	24	1.0 (Ref)	1	1.0 (Ref)
Female	550	38	1.1 (0.7–1.8)	3	0.1 (0.0–0.4) *	18	21.1 (2.8–161.0) *
**Family history of retinoblastoma**							
No/Unknown	891	60	1.0 (Ref)	21	1.0 (Ref)	18	1.0 (Ref)
Yes	237	13	1.1 (0.6–2.1)	6	1.3 (0.5–3.4)	1	0.4 (0.1–3.1)
**Age at RB diagnosis**							
0–11 months	661	39	1.0 (Ref)	12	1.0 (Ref)	9	1.0 (Ref)
12–23 months	303	18	0.7 (0.4–1.3)	9	1.2 (0.5–2.9)	5	0.6 (0.2–1.9)
≥24 months	164	16	1.4 (0.8–2.6)	6	1.9 (0.7–5.2)	5	1.1 (0.3–3.5)
**Year of RB diagnosis**							
1914–1959	304	34	1.0 (Ref)	15	1.0 (Ref)	7	1.0 (Ref)
1960–1969	297	26	0.7 (0.4–1.2)	9	0.5 (0.2–1.2)	7	1.1 (0.4–3.2)
1970–1979	248	10	0.6 (0.3–1.3)	3	0.4 (0.1–1.4)	5	3.0 (0.9–10.0)
1980–2006	279	3	0.5 (0.1–1.6)	0	–	0	−
**Treatment for retinoblastoma**							
Radiation, no chemotherapy	534	33	1.0 (Ref)	12	1.0 (Ref)	5	1.0 (Ref)
Radiation and chemotherapy	435	35	1.4 (0.9–2.3)	13	1.5 (0.7–3.4)	12	2.7 (0.9–8.0)
Other ^†^	159	5	0.6 (0.2–1.5)	2	0.6 (0.1–2.6)	2	1.5 (0.3–8.0)
**Prior malignancy**							
No	932	54	1.0 (Ref)	18	1.0 (Ref)	13	1.0 (Ref)
Yes	196	19	3.5 (2.0–6.1) *	9	3.7 (1.6–8.7) *	6	6.4 (2.2–18.7) *

* Statistically significant *p* < 0.05. ^†^ Includes 16 hereditary survivors treated with radiotherapy but unknown chemotherapy status; the remainder were not reported to have received chemotherapy or radiotherapy (i.e., were treated with surgery alone). ^‡^ Hazard ratios (HRs) and corresponding 95% confidence intervals (CIs) based on multivariable Cox regression model for risk of any benign tumor among hereditary retinoblastoma survivors and for risk of the common benign subtypes lipoma and leiomyoma.

## Data Availability

The data presented in this study are available on request from the corresponding author. In accordance with consent provided by participants, the data are not publicly available.

## Supplementary Materials

The following are available online at https://www.mdpi.com/article/10.3390/cancers13081773/s1, Table S1: Frequency and patterns of benign tumors* among nonhereditary retinoblastoma survivors who reported at least one benign tumor during follow-up.
