# Supplementary material for: Benign Tumors in Long-Term Survivors of Retinoblastoma
Source: Cancers (Basel). 2021 Apr 8;13(8):1773. doi: 10.3390/cancers13081773 (PMC8068196; doi:10.3390/cancers13081773)
Supplement: Supplementary file 1 [file cancers-13-01773-s001.pdf]

cancers MDPI

Supplementary Materials

Benign Tumors in Long-Term Survivors of Retinoblastoma

Milo van Hoefen Wijsard, Sara J. Schonfeld, Flora E. van Leeuwen, Annette C. Moll, Armida W. Fabius, David H. Abramson, Johanna M. Seddon, Jasmine H. Francis, Margaret A. Tucker, Ruth A. Kleinerman and Lindsay M. Morton

Supplementary Materials:

Table S1. Frequency and patterns of benign tumors* among nonhereditary retinoblastoma survivors who reported at least one benign tumor during follow-up.

First Benign Tumor	Second Benign Tumor	Subsequent Benign Tumors	N People
Lipoma			1
Lipoma	Lipoma	3rd Lipoma	1
Leiomyoma			5
Leiomyoma	Leiomyoma		1
Fibroma			1
Osteochondroma			4
Meningioma			2
Fibroadenoma	Fibroadenoma	3rd Fibroadenoma & 4th Leiomyoma	1

*Morphology codes for 22 benign tumors in nonhereditary retinoblastoma survivors; 8 leiomyomas (ICD-O-3 morphology: 8890), 4 lipomas (8850), 4 osteochondromas (9210, 9920), 3 fibroadenomas (9010), 2 meningiomas (9530) and 1 fibroma (9540).

Citation: van Hoefen Wijsard, M.; Schonfeld, J.S.; van Leeuwen, F.E.; Moll, A.C.; Fabius, A.W.; Abramson, D.H.; Seddon, J.M.; Francis, J.H.; Tucker, M.A.; Kleinerman, R.A.; Morton, L.M. Benign Tumors in Long-Term Survivors of Retinoblastoma. *Cancers* **2021**, *13*, 1773. https://doi.org/10.3390/cancers13081773

Academic Editor: Simon Saule

Received: 25 February 2021
Accepted: 2 April 2021
Published: 8 April 2021

Publisher's Note: MDPI stays neutral with regard to jurisdictional claims in published maps and institutional affiliations.

Copyright: © 2021 by the authors. Submitted for possible open access publication under the terms and conditions of the Creative Commons Attribution (CC BY) license (http://creativecommons.org/licenses/by/4.0/).
